# Does high level youth sports participation increase the risk of femoroacetabular impingement? A review of the current literature

**DOI:** 10.1186/s12969-016-0077-5

**Published:** 2016-03-11

**Authors:** Viran de Silva, Michael Swain, Carolyn Broderick, Damien McKay

**Affiliations:** Children’s Hospital Institute of Sports Medicine, Locked Bag 4001, Westmead, Sydney, New South Wales 2145 Australia; Territory Sports Medicine, Darwin, Northern Territory Australia; The George Institute for Global Health, Sydney Medical School, University of Sydney, Sydney, Australia; Department of Chiropractic, Faculty of Science, Macquarie University, Sydney, 2109 Australia; Children’s Hospital Institute of Sports Medicine, The Sydney Children’s Hospitals Network, Sydney, Australia; School of Medical Sciences, UNSW Medicine, University of New South Wales, Sydney, Australia

**Keywords:** Femoroacetabular impingement, Adolescent, Sports Participation

## Abstract

Sports participation can be an integral part of adolescent development with numerous positive short and long-term effects. Despite these potential benefits very high levels of physical activity, during skeletal maturation, have been proposed as a possible cause of cam-type femoroacetabular impingement (FAI). The influence of physical activity on the developing physis has been previously described both in animal studies and epidemiological studies of adolescent athletes. It is therefore important to determine whether the development of FAI is secondary to excessive physical activity or a combination of a vulnerable physis and a set level of physical activity. A review of the current literature suggests that adolescent males participating in ice-hockey, basketball and soccer, training at least three times a week, are at greater risk than their non-athletic counterparts of developing the femoral head-neck deformity associated with femoroacetabular impingement.

## Background

Femoracetabular impingement (FAI) was first described in 1936 by Smith-Peterson before Ganz et al. in 2003 proposed FAI as an aetiological factor for the development of early osteoarthritis (OA) in non-dysplastic hips [1]. Recent evidence suggests that FAI is not only a cause of premature osteoarthritis but also a cause of hip pain and reduced activity both in young adults and athletically active individuals, including elite athletes [2, 3].

Femoroacetabular impingement is a condition of abnormal contact between the proximal femur and the acetabulum, secondary to either abnormal bony morphology or excessive range of motion in those with seemingly normal anatomy [1]. The two surfaces do not come into contact in normal physiological range but do so in FAI [4]. FAI can be divided into two major sub-groups—pincer and cam-type deformity, with 50–70% of patients having evidence of both forms of impingement [5].

**Fig. 1:**
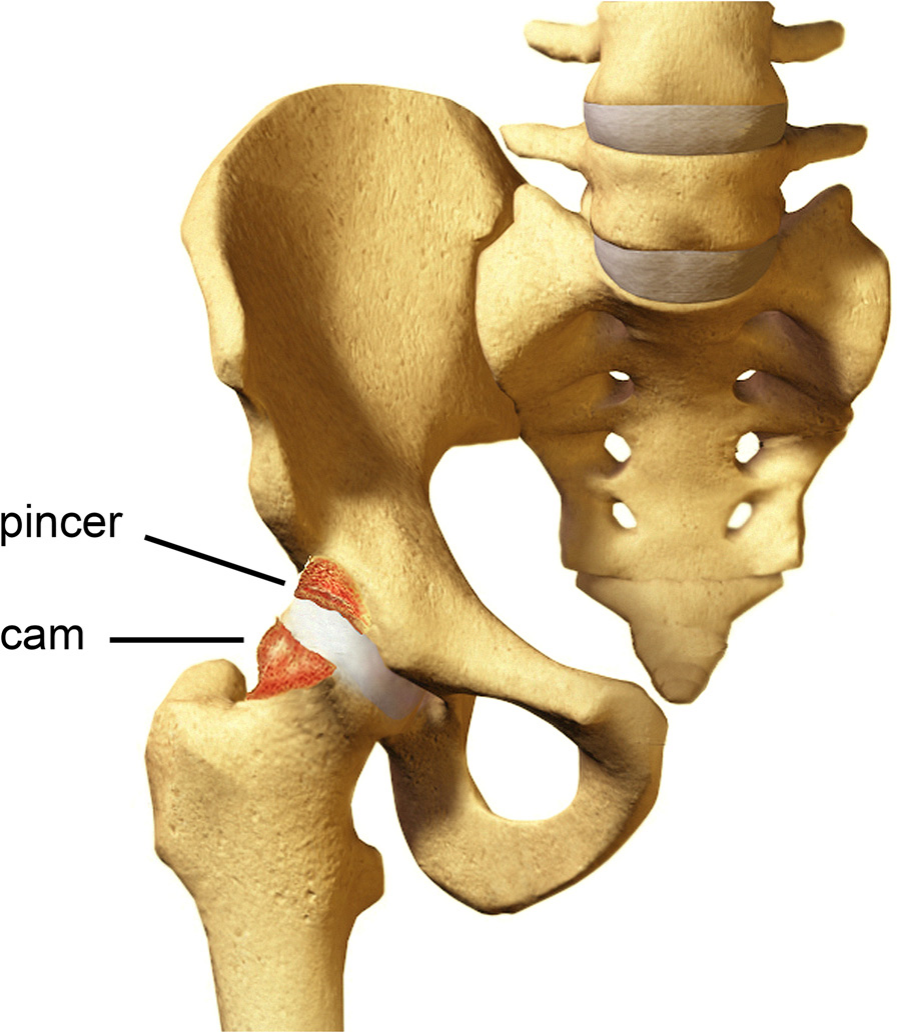
Anatomical changes of FAI. Acetabular over-coverage leading to pincer deformity. Abnormal bony morphology of the head neck junction leading to cam deformity

In pincer type FAI the pathology is in the acetabulum caused by an increased acetabular depth resulting in acetabular overcoverage (Fig. 1) [5, 6]. During hip flexion the femoral head-neck repeatedly abuts the acetabulum. The repeated microtrauma to the anterior acetabular margins causes labral tears and articular cartilage damage, eventually leading to OA [4, 7]. In cam-type FAI the abnormality is an aspherical femoral head as well as reduced cranial offset of the femoral head-neck junction, often secondary to extra bone formation (Fig. 1) [4, 6]. Like pincer type FAI, flexion and internal rotation of the hip results in repetitive impingement of the prominent femoral head and acetabulum, leading to labral tearing, cartilage delamination and ultimately OA [8].

Conditions resulting in morphological changes of the hip including slipped capital femoral epiphyses (SCFE), Legg-Calve-Perthes Disease, coxa vara, and osteonecrosis can contribute to the development of FAI [6, 9]. It is now apparent that the development of FAI may also be due to developmental adaptations during skeletal maturation as a result of activities that involve repetitive hip motion [10]. Recent reports document an increased prevalence of FAI type-deformities in elite adolescent athletes compared to their age-matched non-athletic controls [11]. This suggests that the morphological changes of FAI may be a response to repetitive stress at the proximal femoral physis secondary to sporting activity during periods of skeletal growth [2, 12]. This would further imply that chronic mechanical stress during growth is an aetiological factor for abnormal hip shape and early hip joint degeneration.

Evidence suggests that FAI is commonly missed in adults and likely to be missed when present in adolescents, where patients may often present initially [13]. Early recognition by pediatric rheumatologists is required to differentiate FAI from an inflammatory arthropathy. The condition is potentially under-recognised in pediatric rheumatology clinics, where clinicians are well positioned to have meaningful impact on disease progression through early advice on activity levels.

While much is known about FAI with respect to the affected population, presentation, potential interventions and outcomes, the precise cause of the abnormal morphology that results in FAI and its relationship to sports participation in adolescents remains unclear. This is the focus of this review.

## Methods

A review of the literature was performed in January 2015 to identify articles in which the prevalence of FAI was reported in athletically active child and adolescent groups. The computerized databases PubMed, CINAHL, Medline (Ovid) and EMBASE were searched for primary studies from the earliest year possible to 31st January 2015. Hand searches of the reference lists of all included publications were checked for potentially relevant articles and citation tracking (backwards and forwards) was also performed. Search terms used included child, adolescent, hip impingement, femoroacetabular impingement, CAM deformity, Pincer deformity and physical activity.

Studies were reviewed based on the following eligibility criteria: Epidemiological studies with prospective cohort, cross sectional or case–control designs; with participants whose mean age or age range was between 6 and 25 years where a proportion of participants have been diagnosed with FAI and had regular exposure to organized physical activity. Finally, included studies needed to report the prevalence of FAI in the context of a study population setting. Exclusion criteria included retrospective studies and case reports, case-series with <10 participants, commentary papers, literature reviews and studies that evaluate the outcome of a clinical intervention for FAI.

## Results

**Fig. 2:**
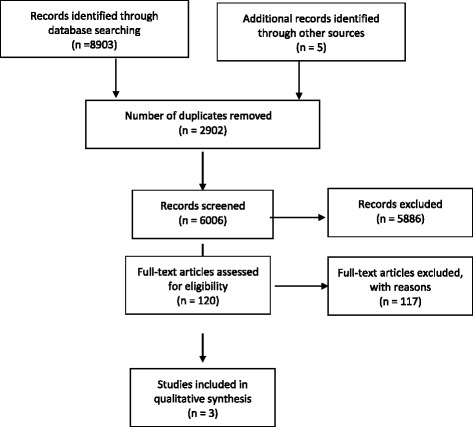
Flow diagram. Flow diagram of search strategy

This search yielded 8903 studies (Fig. 2). In addition, the “related citations” feature of PubMed and the reference list from each study were searched to determine whether any relevant studies had been missed by the search strategy. This yielded a further 5 studies. After duplicates were removed from the initial search, citation titles and abstracts were independently screened for relevance by two review authors (VdS and MS) in accordance with the predetermined eligibility criteria. 120 full-text articles were assessed for eligibility with 3 studies meeting exact criteria, and are included in Table 1.

**Table 1:** Study Characteristics

Study	Study Design	Study Size	Study Population	Age (range, mean SD)	FAI Type	Method of Diagnosis	Study definition of FAI
Siebenrock 2011 [12]	Case Control	75	37 Elite Male Basketball players and 38 non-athletic controls	Age 9–25 years (mean 17.6)	cam-type deformity	MRI	Alpha angle of >55° from the 9 o’clock (posterior) to the 3 o’clock positions
Philippon, 2013 [14]	Cohort	88	61 amateur male ice hockey player and 27 skiers	Age 10–18 years (mean 14.5 hockey players) (mean 15.2 skiers)	cam-type deformity	MRI	Alpha angle of >55°
Agricola, 2014 [15]	Cohort with 2 year follow up	63	63 Elite Male soccer players	Age 12–19 years (mean 14.4)	cam-type deformity	X-ray	Alpha angle >60

Siebenrock et al. in a comparison of adolescent basketball players from a local professional club aged between 9 and 25 (mean 17.6 years) with aged matched controls demonstrated that after physeal closure, 89 % of athletes had an alpha angle greater than 55° compared with 9 % in the control group [12] (Table 2). In addition, a greater number of athletes had increased alpha angles after physeal closure than before growth plate closure. This is compared to the control group, which showed no change in mean alpha angles before and after growth plate closure [12].

**Table 2:** Prevalence of CAM deformity in included studies

Study	Population	Prevalence of CAM deformity		Significance
		Athletes	Non-Athlete	
Siebenrock, 2011 [12]				
	Open Physis	N/A	N/A	
	Closed Physis	89 %	9 %	*P* = 0.001
Philippon, 2013 [14]				
	Pewee (10–12years)	37 %	43 %	
	Bantam (13–15years)	63 %	63 %	
	Midget (16–19years)	93 %	25 %	
	Overall	75 %	42 %	*p* < 0.006
Agricola, 2014 [15]				
	Alpha-angle	38 %		*p =* 0.51
	Flattening of HNJ	47 %		*p =* 0.46
	Prominence of HNJ	22 %		*p* < 0.001

*HNJ* Head Neck Junction

Phillipon demonstrated significantly higher alpha angles in a group of high-level ice-hockey athletes compared to a group of skiers (control). 46 athletes from a cohort of 88 (52 %) met the study definition of FAI, compared with 11 (12 %) from the control group. (Table 3) Further to this, 75 % of the ice-hockey group had an alpha angle greater than 55°, compared with 42 % in the skier group (*P* < 0.006) [14]. (Table 2)

**Table 3:** Study Variables

Study	Total number of hips with FAI	Total number of hips in Study	Total number of participants with FAI	Total number of participants	Total number of controls with FAI	Total number of controls	Total number of sports participants with FAI	Total number of sports participants
Siebenrock 2011 [12]	N/A	148	N/A	74	N/A	N/A	N/A	N/A
Philippon 2013 [14]	57	176	57	88	11	27	46	61
Agricola 2014 [15]	49	126	N/A	63	N/A	0	N/A	63

When the ice-hockey players were divided into age groups, those in the midget group (mean age 17.4 years) with complete physeal closure had an increased prevalence (93 %) of a raised alpha angle. This is compared not only to their age matched skier controls, but also to ice hockey players in the bantam-level (mean age 14.6) and peewee-level (mean age 11.7) groups, perhaps suggesting that the likelihood of having a high alpha angle increases with age, but also with level of play [14]. The prevalence of alpha angle greater than 55° in the bantam and pewee aged hockey players was 63 % and 37 % respectively, though this was not significant when compared to their own aged match controls [14].

Agricola, in a 2-year follow up study of elite adolescent soccer players noted the prevalence of a prominence at the head neck junction in the entire soccer group to increase significantly from 7.1 to 22.2 % at follow up. In the hips with an open growth plate at baseline the prevalence of a prominence increased significantly from 2.1 to 17.7 %. After physeal closure there did not appear to be a significant increase in the prevalence of a cam deformity in the soccer players [15].

## Discussion

**Fig. 3:**
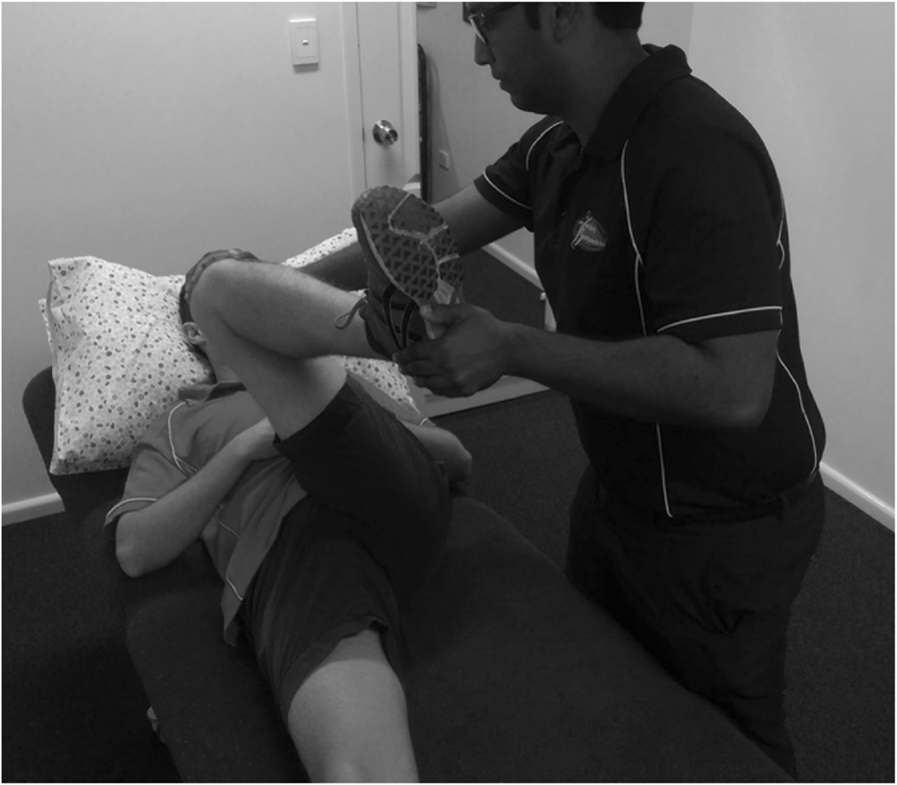
Clinical Examination. Flexion, Adduction and Internal Rotation (FADDIR) of the hip reproducing symptoms

Clinically, patients with FAI often present with an insidious onset of anterior groin pain, made worse with activities involving hip flexion. Examination reveals limited terminal hip range of motion and positive anterior impingement testing, by way of reproducible hip pain with passive hip flexion, adduction and internal rotation (Fig. 3) [16–19].

Radiologically, consensus is yet to be reached on how best to confirm the presence of cam or pincer impingement. Plain radiographs of an antero-posterior (AP) pelvis, frog leg lateral and 45**°** or 90**°** Dunne view, can ascertain the presence of coxa vara, femoral retroversion or a prominence in the anterolateral femoral head (femoral head-neck junction) to suggest cam impingement. Evidence of acetabular overcoverage, such as coxa profunda, protrusio acetabuli or femoral head deformity may indicate pincer impingement [16, 18, 20]. Alternatively, magnetic resonance imaging (MRI) is now perhaps the most popular mode of investigation [12]. The α angle, measured on both plain radiographs and MRI, is the angle between the femoral neck axis and a line connecting the centre of the femoral head with a point along the head-neck contour where it becomes aspheric. It is used as a marker for assessing the femoral head-neck contour, with a greater angle representing a more pronounced aspherical shape of the femoral head [3, 21]. Notzli et al. in their original description proposed an angle greater than 50° when using MRI as a marker of an abnormally shaped femoral head-neck contour [22]. Contrastingly, further work by Agricola, Waarsing et al. has suggested an α angle threshold of 60° for the presence of a cam deformity when using plain radiographs, with a pathological threshold of 78° for the likely development of OA [23]. Additional studies have also quoted different values between sexes, suggesting a maximal normal α angle of 68° in men and 50° in women [16]. When using MRI, many studies suggest a positive alpha angle for values greater than 55° [12, 14]. Consequently, FAI continues to remain a clinical diagnosis with no obvious pathognomic value for the α angle as yet.

Multiple potential etiologies for the development of FAI have been proposed including intrinsic genetic factors, long term consequences of pediatric hip disease and femoral neck fracture, post surgical correction and activity-related developmental factors [10]. The increased prevalence of FAI in athletic cohorts compared to their non-athletic controls in both adults and adolescents would suggest that the development of FAI is, at least in part, associated with abnormal or excessive loading [15, 24].

Murray and Duncan in 1971 noted an increased prevalence of what was then called a “femoral head tilt deformity” in adolescents who participated in a school based sports program compared to their controls [25]. Since then it has been theorised that abnormal exertion during the adolescent growth period, is a risk factor for the development of cam impingement [25].

The epiphyseal growth plate is a dynamic entity during adolescence [26]. It is responsible for longitudinal bone growth and is regulated by a complex interaction of hormones and growth factors [27]. During the adolescent growth spurt there is increased growth plate width and activity, both of which gradually decline once skeletal maturity is reached [26, 28].

The developing skeleton is more responsive than the mature skeleton to the osteotrophic effects of exercise and shows more pronounced adaptive changes to intense sports training [29–31]. Despite this, and their greater remodeling potential when compared with adults, adolescent growth plates are often less resistant to deforming forces than ligaments and joints [26]. Similarly, adolescents and children who participate in high intensity, regular sporting activity are prone to repetitive loads and trauma. This repetitive trauma can alter metaphyseal perfusion resulting in a spectrum of injuries ranging from osseous necrosis to growth disturbance [32].

Results from animal studies have also found excessive repetitive physical loading to be an aetiological factor for physeal stress related injuries. A number of case reports or case series have been published reporting physeal injuries of the distal radius in gymnasts, and stress related changes in the physis in adolescent baseball pitchers as a result of excessive load (pitching) [33–37]

### Amount of activity performed

In their study on FAI, Carson et al. used the Habitual Activity Estimation Scale (HAES) to quantify the amount of activity performed by the athletic group and noted a significant difference in the amount of hours performed in individuals with cam deformity compared to the control group, particularly on weekends [38]. Siebenrock defined basketball participation as being continuous involvement in club training and games since the age of 8. This would evolve from three training sessions and games for 9–12 year olds, four to five training sessions and games for 13–15 year old, and eight training sessions or games per week for athletes 16 years and older [12]. In ice-hockey, athlete participation ranged from a minimum of 1 year participation with training sessions varying between 3 and 8 times a week increasing with age, to competing for 38 +/− 12.5 weeks per year [14, 39]. Similar activity levels were noted in soccer players, with those training 4 times a week or greater at professional club before the age of 12 having a significantly higher prevalence of cam deformity as an adult [40].

### Further studies

In a study of 44 volunteers (88 hips) 14 % of particpants had at least one hip with an alpha angle greater than the defined 50.5°, contrasting to 0 % of patients with an open physis [38]. Whilst none of the volunteers played any one particular sport, analysis of their physical activity levels using the HAES, a validated pediatric activity score, demonstrated that those positive for cam morphology were “very active” on Saturdays with 7.1 (+/− 1.59 SE) hours of activity, compared with 2.9 h (+/− 0.51SE) in those with alpha angles less than 50.5° [38].

Additional work by Siebenrock et al. in adolescent ice hockey players revealed 56 % of athletes with a closed physis had an alpha angle greater than 55° [39]. Again, they also demonstrated an increase in alpha angle from a seemingly normal range to an abnormal range following physeal closure, replicating their observation in the basketball players [39].

Agricola et al. in their original study of adolescent soccer players reported 26 % of athletes had an alpha angle greater than 60° on plain radiographs. This compared with 17 % of controls, which was not significant when corrected for age. A flattening of the head-neck junction, assessed as being a moderate decrease in the anterior head-neck offset compared to the posterior head-neck junction was however significantly more frequent in soccer players than controls (53–19 % *P <* 0.001) [4].

A prospective study of 67 males by Kapron et al. used x-rays and an alpha angle of greater than 50**°** to determine the prevalence of FAI in collegiate footballers. They noted 95 % of the 134 hips included for analysis had at least 1 radiographic sign of CAM or pincer impingement [5]. Seventy two percent of athletes had an abnormal alpha angle on either AP or frog-leg lateral radiograph [5]. Larson et al. noted a similarly high prevalence of radiographic FAI in a separate cohort of male collegiate football players. In their study, 75 % of athletes met the definition for radiographic CAM impingement with an alpha angle greater than 55**°** [41].

Perhaps tellingly in a recent meta-analysis, including many of the above studies, the pooled prevalence rate by hip for cam deformity in male athletes was 41 % compared with 17 % for male controls (*P <* 0.001) [42]. The pooled prevalence rate for cam deformity in individual male athletes was 29 % compared with 19 % for male controls (*P* = 0.02). They determined that athletes participating in sports during adolescence, namely ice-hockey, basketball and jumping sports, are at a 1.9 to 8 times increased risk of developing a cam deformity during skeletal maturation [42]. Unsurprisingly, youth ice hockey players have been shown to repetitively internally rotate with hip flexion during the recovery phase of the hockey stride, replicating the known biomechanical positions that lead to hip impingement [43].

When comparing the prevalence of CAM impingement against the general adolescent population, the prevalence was found to range from 6.1 % in radiographic studies through to 24 % in a study of asymptomatic young Swiss males presenting for conscription [44]. In the asymptomatic adult population Gosvig et al. studying 4151 adults as part of the Copenhagen Osteoarthritis Substudy revealed the prevalence of cam impingement to be 17 % in males and 4 % in females using radiographic assessment [8]. In their study, however, they used pathological alpha angles of 83**°**for males and 57**°** for females [8]. Further studies though using an alpha angle of 50.5**°** have shown a similar prevalence of 14 % in 200 asymptomatic volunteers whilst the prevalence of radiographic findings consistent with CAM impingement in a healthy population of 2081 adult patients ranged from 3.3 % in female patients to 21.5 % in male patients [44, 45].

Physeal stress has been proposed as a cause of cam impingement, and the association between physical activity and its influence on the developing physis has been established at other sites. The relative contributions of loading and physeal vulnerability in the development of FAI however remain unknown, which makes it difficult to predict, how much is too much?

## Conclusion

Femoroacetabular impingement is a recognised cause of hip pain and reduced activity, particularly in adolescents and young adults. Multiple aetiologies have been proposed by recent epidemiological studies and an understanding of skeletal maturation suggests that excessive activity during the adolescent growth period may play a key role in the development of FAI. In particular, adolescent males who participate in ice-hockey, basketball and to a lesser extent soccer, whilst performing a minimum of three training sessions and games per week are currently at greatest risk of developing a cam deformity and potentially progressing to symptomatic hip impingement. Further research by way of longitudinal studies or a systematic review looking at the comparison between sporting participation and a control cohort in athletes with open physis is required.

Identified prevalence data in the current literature suggests that highly regular activity during adolescent development, particularly in the presence of an open physis can encourage the development of a cam deformity. Similarly, sports that require repetitive high impact loading, hip flexion and internal rotation such as basketball, ice hockey and soccer employ movement patterns most likely to contribute to cam development.

Pediatric rheumatologists have an important role in identifying and diagnosing at risk individuals with femoroacetabular impingement, particularly given the potentially long-term consequences.

## Abbreviations

AP: Antero-posterior
FAI: Femoroacetabular impingement
HAES: Habitual activity estimation scale
HNJ: Head neck junction
MRI: Magnetic resonance imaging
OA: Osteoarthritis
SCFE: Slipped capital femoral epiphysis
